# Clinicopathological Profile and Management of Appendiceal Neoplasm: An Observational Study

**DOI:** 10.31729/jnma.63.290.9206

**Published:** 2025-09-01

**Authors:** Abhishek Bhattarai, Prasan Bir Singh Kansakar, Laligen Awale, Bikal Ghimire, Homendra Kumar Sah

**Affiliations:** 1 Department of General Surgery, Maharajgunj Medical Campus, Institute of Medicine, Mahrajgunj, Kathmandu, Nepal

**Keywords:** *adenocarcinoma*, *appendectomy*, *appendiceal neoplasm*, *mucinous neoplasm*, *neuroendocrine tumor*

## Abstract

**Introduction:**

Appendiceal neoplasms are rare and frequently diagnosed incidentally during histopathological examination of appendectomy specimens. Recent data suggest that their incidence is increasing worldwide. This study aimed to evaluate the appendiceal neoplasm in appendectomy specimens, determine their clinicopathological presentation, histological subtypes, diagnostic modalities used, and the outcomes at the Tribhuvan University Teaching Hospital, Kathmandu, Nepal.

**Methods:**

A retrospective evaluation of patients diagnosed with appendiceal neoplasm was conducted from January 2013 to December 2023 at Tribhuvan University Teaching Hospital in Kathmandu, Nepal. Ethical approval was obtained from the Institutional Review Board (Ethical Approval Reference No: 32081/082). Various parameters, including demographic profiles, disease pathology and outcomes were studied.

**Results:**

A total of 57 cases of appendiceal neoplasm were identified during the study period. The mean age at diagnosis was 47.1±18.5 years. Among them, 29 (50.87%) were male and 28 (49.12%) were female. Of the total cases, 52 (91.22%) cases were primary appendiceal neoplasms, 3 (5.26%) were secondary, and 2 (3.50%) cases remained unclassified. The primary mucinous appendiceal neoplasm was seen in 43 (75.44%), followed by neuroendocrine tumor in 5 (8.77%) cases. Appendectomy was performed in 29 (50.88%) and right hemicolectomy in 15 (26.32%) cases. The major perioperative complications were observed in 2 (3.50%) cases, with 1 (1.75%) case of mortality.

**Conclusions:**

The number of diagnosed appendiceal neoplasms increased over the year with mucinous tumors being predominant followed by neuroendocrine tumor. Appendectomy was adequate in most cases, with limited need for further surgery, and outcomes remain excellent despite few of them developed major postoperative complications.

## INTRODUCTION

Appendiceal neoplasm (AN) are rare and most often diagnosed during histopathological examinations (HPE) of appendectomy specimens or surgery.^1^ Occasionally, they can be identified pre-operatively as masses in the right iliac fossa (RIF).^2^ The appendix, despite its small size, can develop different types of cancer, each with unique biological and histological characteristics.^3^ Due to the rarity of these conditions, the natural history of each subtype is poorly understood, and several management options have been proposed in the literature.^4,5^

In Nepal, the natural history and epidemiology of ANs is unknown. The incidence and histopathological types of ANs vary significantly across different nations.^6-8^ Reports from various international studies indicate a trend of increasing incidence of ANs in the last 30 years.^9-11^

Therefore, our aim is to evaluate the appendiceal neoplasm in appendectomy specimens, assess their clinicopathological presentation, histological subtypes, diagnostic modalities used, and the outcomes associated with these conditions.

## METHODS

This retrospective, single-center observational study included all patients diagnosed with AN between January 2013 to December 2023 at the Department of General Surgery, Tribhuvan University Teaching Hospital in Kathmandu, Nepal. Ethical approval was obtained from the Institutional Review Board (Ethical Approval Reference No: 32-081/082).

Non-probability consecutive sampling technique applied for the above-mentioned study duration. The retrospective data was collected from departmental database, encompassing all appendectomy specimens evaluated with histopathology at our center during study period. The study included all appendectomy cases, both elective and emergency procedures, and spanned all age groups, from pediatric to adult patients. All the cases diagnosed with AN in HPE reports (with or without immunohistochemical staining) were included in the study. However, the cases with missing detailed HPE report, inadequate imaging and operative details were further excluded from the study.

These patient details were recorded using a pre-formed proforma that included demographics, mode of presentation, radiological imaging (ultrasound/CT scan), surgical approach, intraoperative observations, HPE results, postoperative course, and oncological follow-up. Specific attention was given to the completion of surgical treatment if required. Based on the histological diagnosis, neoplasms were categorized and compared with the assessed parameters.

The data were analyzed using the Statistical Package for Social Sciences SPSS for Windows, version 26 (IBM Corp., Armonk, N.Y., USA). Results are presented as numbers and percentages in tabular or graphical form. Continuous variables are reported as either the Mean SD for symmetric and non-skewd Median for skewed mean. Categorical variables are reported as numbers and proportions. Histological subtypes and surgical management are presented in tabular format.

## RESULTS

**Figure 1:**
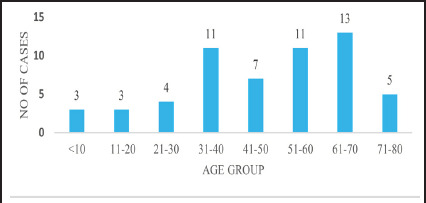
Age distribution of the patients with appendiceal neoplasm (n=57).

**Figure 2:**
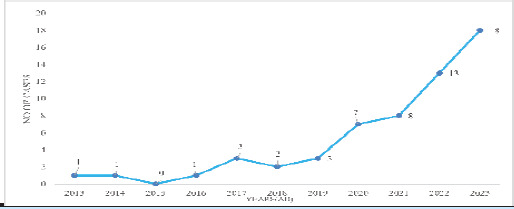
Annual incidence of appendiceal neoplasm per year (n=57).

Among all the patients who underwent histopathological evaluation for appendiceal pathology during the study period, 57 cases were identified as ANs and included in the study while the rest were excluded. Of these, 29 (50.87%) were male and 28 (49.12%) were female. The mean age at diagnosis was 47.1 years, with an SD ±18.5 years. There were 13 cases of ANs in 61-70 years of age group and 11 cases in 31-40 years and 51-60 years of age group (Figure 1). In 2023, 18 cases of appendiceal neoplasm were observed (Figure 2).

Out of total 57 cases, 25 (43.85%) cases presented as acute appendicitis. 43 (75.44%) cases were mucinous neoplasm followed by neuroendocrine tumors in 5 (8.77%) cases (Table 1). The clinical and histopathological characteristics of patients with NETs (Table 2).

**Table 1:** Demographic, Clinico-pathological profile and management of appendiceal neoplasm (n=57).

Category / Variable	n(%)
**Mode of presentation**	
Acute appendicitis	25(43.85)
Mass in RIF	20(35.09)
Chronic RIF pain	18(31.58)
Incidental diagnosis	7(12.28)
Appendicular lump	6(10.53)
Intestinal obstruction	3(5.26)
Right upper quadrant pain	1(1.75)
**Diagnosis**	
Pre-operative	27(47.37)
Intra-operative	11(19.30)
Post-operative	19(33.33)
**USG findings (n = 55)**	
Appendicitis	22(40.00)
Mucocele/Neoplasm	17(30.91)
Mesenteric cyst	1(1.82)
Inconclusive	15(27.27)
**CT scan findings (n = 37)**	
Appendiceal neoplasm	24(64.86)
Other pathology	13(35.14)
**Histology**	
Mucinous neoplasm	43(75.44)
Low grade mucinous neoplasm (LAMN)	39(90.70)
High grade mucinous neoplasm (HAMN)	2(4.65)
Mucinous adenocarcinoma	2(4.65)
Neuroendocrine tumor (NET)	5(8.77)
Adenocarcinoma	5(8.77)
Primary (Classical colonic type)	3(60.00)
Secondary	2(40.00)
Lymphoma	3(5.26)
Primary (Diffuse large B cell type)	1(33.33)
Unclassified	2(66.66)
Marginal zone	1(33.33)
Anaplastic	1(33.33)
Adeno-neuroendocrine neoplasm (Secondary)	1(1.75)
**Operative resection**	
Appendectomy	29(50.88)
Right hemicolectomy	15(26.32)
Ileocecal resection	10(17.54)
Appendectomy followed by Right hemicolectomy	3(5.26)
**Chemotherapy**	
Neoadjuvant chemotherapy	1(1.75)
Adjuvant chemotherapy	9(15.79)

**Table 2:** Clinical and histopathological characteristics of patients with Neuroendocrime tumours (n=5).

Age/Sex	Location	Size(cm)	Infiltration	Vascular Invasion	Perineural Invasion	Lymph node	Differentiation	Grade	Mitosis	Ki-67
42/F	Tip	1.5x1	Subserosa	Absent	Absent	Absent	Well	G2	2-20	<3
14/M	Tip	0.5x0.5	Muscularis	Absent	Absent	Absent	Well	G1	<2	<3
30/F	Tip	0.8x0.7	Muscularis	Absent	Absent	Absent	Well	G1	<2	<3
40/M	Base	1.2x1	Subserosa	Absent	Absent	Absent	Well	G1	<2	<3
18/F	Tip	0.9x0.6	Muscularis	Absent	Absent	Absent	Well	G1	<2	<3

Out of total 57 cases, 25 (43.85%) cases presented as acute appendicitis. 43 (75.44%) cases were mucinous neoplasm followed by neuroendocrine tumors in 5 (8.77%) cases. Computed tomography (CT) scan successfully detected ANs in 24 (64.86%) cases pre-operatively. In the remaining 13 (22.81%) cases, other pathology was evident, among which ileocecal mass was diagnosed in 5 (38.46%) cases. CT scan was conducted postoperatively in 2 (3.51%) cases. Intraoperative suspicion led to extended resections in 5 (8.77%) cases, while 19 (33.33%) cases of ANs were diagnosed post-operatively on HPE following surgery for presumed appendicitis or other pathology (Table 1). The clinical and histopathological characteristics of patients with NETs (Table 2).

Of the total ANs, 52 (91.22%) were primary, 3 (5.26%) were secondary, and 2 (3.50%) cases remained unclassified. In total, 29 (50.88%) cases were managed with appendectomy as the primary surgical intervention. Postoperative complications were observed in a total of 17 (29.82%) cases including surgical site infections in 16 (28.07%) cases, chest infections in 8 (14.03%) cases, anastomotic leaks in 2 (3.50%) cases and postoperative bowel obstruction in 2 (3.50%) cases. The major surgical complications with Clavien-Dindo grade ≥3 were observed in 2 (3.50%) cases and there was one case of perioperative mortality. The median duration of hospital stay was 3 days.

## DISCUSSION

Demographic data from different countries show a rising annual incidence of ANs, with incidence rates ranging from 0.2% to 3.2%.^11-14^ A significant rise in case numbers was observed in our study following 2020. This increase may be attributed to shifts in population demographics, enhanced availability of diagnostic and histopathological services, greater awareness and reporting, evolving management approaches, and revisions in pathological grading and staging criteria.^15^

ANs were identified across all age groups, with 50% of cases occurring in individuals over the age of 50.^16,17^ This age-related pattern is further supported by a Turkish multicenter study, which demonstrated a rising odds ratio for ANs with increasing age, peaking in patients aged 70 and above—indicating that age may be a potential risk factor for ANs.^15^ In our study, the mean age at diagnosis was 47.1 years, with range spanning from 3 to 77 years and few cases were observed in patients under 30 years of age. A similar mean age of presentation was seen in different studies.^12,18^ This pattern highlights a tendency for ANs to present more frequently in middle-aged and older adults, with relatively fewer cases occurring in younger populations. Interestingly, in our cohort, 10 (17.54%) cases were under 30 years, with 6 (60.00%) cases of primarily LAMNs, 3 (30.00%) cases of NETs and 1 (10.00%) case of lymphoma differing from reports that highlight NETs as the most frequent neoplasm in young patients.^19^ Thus, ANs should be considered as a differential diagnosis even in the younger age group.

The clinical presentation of patients with ANs can be variable and nonspecific.^20^ Typically, these patients exhibit symptoms similar to those of acute appendicitis.^21^ Some of them experience the right lower quadrant pain and a palpable mass in the RIF.^21^ In some cases, patients may present with intestinal obstruction and intussusception.^22,23^ Additionally, some neoplasms are discovered incidentally during HPE.^20^ In our cohort also, 25 (43.85%) cases presented with symptoms of acute appendicitis, while 20 (35.09%) cases presented with RIF mass and 18 (31.58%) cases with chronic RIF pain. Similarly, 3 (5.26%) cases presented with intestinal obstruction, one of which was due to recurrent intussusception. This accounted for the high rate of appendectomies in 29 (50.88%) cases while, 19 (33.3%) cases were incidentally diagnosed during HPE.

Preoperative diagnosis remains challenging, particularly for tumors confined to the appendix. CT scan is the preferred modality to diagnose the disease when an appendiceal mass is suspected.^21,24^ However, small or asymptomatic tumors like NETs often evade detection by CT scan also.^21^ In our study, the diagnostic accuracy was 30.91% for USG and 64.86% for CT scan, signifying the need of imaging in patients with suspected appendicitis. However, none of the imaging was able to identify NETs, adenocarcinoma or lymphoma pre-operatively.

Histologically, ANs are mainly divided into five main histopathologic subtypes: neuroendocrine tumors (NET) as nonepithelial tumor, while mucinous neoplasms, colonic-type (non-mucinous) adenocarcinomas, goblet cell adenocarcinomas (GCA) and signet ring cell adenocarcinomas as epithelial tumors.^25,26^ Histological subtypes of appendiceal neoplasm vary globally. Some centers report a predominance of NETs, while others note higher rates of epithelial tumors. ^9,14,19,27^ Recent studies show a global shift toward epithelial types, with Marmor et al. noting increased epithelial tumors without a drop in NETs incidence, and Naar et al. confirming epithelial predominance in a large cohort.^9,27^ Our findings mirror this, with mucinous neoplasms comprising 43 (75.44%) of cases, followed by 5 (8.77%) of NETs cases.

Epidemiological studies shows that the appendix is the third most common site for NETs in the gastrointestinal tract, following the small intestine and rectum.^28^ These tumors are often discovered incidentally during appendectomy performed for acute appendicitis.^1,28^ They are most commonly located at the tip of the appendix, are less than 2 cm at diagnosis, and associated with a favorable prognosis.^1,28^ Resonating the literature, in this study also, in four out of five cases of NETs, tumor was located at the tip of the appendix, and all measured less than 2 cm in size. Surgical management in NETs should be individualized; however, appendectomy alone is generally sufficient for well-differentiated tumors smaller than 2 cm without mesoappendiceal invasion.^1,28^ In this cohort, all five were well-differentiated, with four being G1 and one G2. As suggested in the literature, appendectomy was sufficient in four cases of our study while the one case with lesion at the base of appendix required revision hemicolectomy. Only one case was suspected intraoperatively, with the rest diagnosed on HPE. None showed vascular or perineural invasion.

Mucinous neoplasms comprise a heterogeneous group of diseases with varying malignant potential. They may present as an unruptured, mucin-filled appendix, or with peritoneal metastases following rupture of the primary tumor.^20,26,29^ Corresponding with existing literature, complications among 43 mucinous neoplasms in our study included localized perforation in 4 (9.30%) cases, intraoperative spillage in 2 (4.60%) cases, and positive appendiceal margins in 2 (4.60%) cases, indicating the risk of peritoneal contamination and incomplete resection. LAMN was predominant pathology among mucinous neoplasm in our study. The surgical management of this types of neoplasm should also be individualized.^1^ Showing conceptual alignment, among 39 cases of LAMNs, 20 (51.28%) patients underwent appendectomy, 7 (17.94%) had ileocecal resection, and 10 (25.64%) underwent right hemicolectomy.

As per the guidelines, appendectomy with long-term follow-up should be sufficient for patients with HAMN with negative margins.^1^ Owing to it, two cases of HAMN, diagnosed postoperatively, were managed with appendectomy and regular follow-up due to negative resection margin. There were 2 (3.50%) cases of mucinous adenocarcinoma including one with pseudomyxoma peritonei and a high Peritoneal Carcinomatosis Index (PCI >30) and one underwent revision surgery after postoperative diagnosis.

Primary colonic-type adenocarcinoma of the appendix is a rare entity, accounting for less than 0.5% of all gastrointestinal neoplasms.^1^ They are also found incidentally following appendectomy for appendicitis.^1^ Currently, there is no specific staging system or established guidelines for its management; therefore, these cases are typically treated in accordance with the protocols for right-sided colonic carcinoma.^1,26^ In our study also, three cases of classical colonic-type adenocarcinoma of the appendix were identified. One case originated from a pre-existing polyp located at the base of the appendix, while the remaining two were diagnosed incidentally following appendectomy.

Lymphoma that are typically confined to the appendix are the primary appendiceal lymphoma. They are very rare. Most of them are non Hodgkins lymphoma. The optimal management of these tumors is thought to involve a multimodal approach, including chemotherapy, surgical resection, radiotherapy, and immunotherapy. However, available data remain insufficient to establish a definitive treatment strategy.^30,31^ In our study, we identified a single case of primary appendiceal lymphoma of the diffuse large B-cell type, presenting with recurrent ileocecal intussusception and intestinal obstruction, which was managed with a right hemicolectomy.

Surgical management depends on histology, disease extent, and preoperative findings.^1,3^ Simple appendectomy suffices for early-stage tumors (LAMN, HAMN, NET <1 cm), aggressive subtypes like mucinous or colonic adenocarcinoma and GCC warrant right hemicolectomy with lymph node dissection.^1,3^ Cytoreductive Surgery (CRS) and Hyperthermic Intraperitoneal Chemotherapy (HIPEC) are preferred for disseminated disease.^26^ Although most of our cases were managed appropriately, 6 (15.38) cases of complicated LAMN could not undergo CRS/ HIPEC due to lack of facilities at our center. Two cases (5.12%) of uncomplicated LAMN diagnosed postoperatively with positive resection margin were kept on regular follow-up and did not show any signs of recurrence for 1 year. It aligns with other studies stating that uncomplicated LAMN confined to the appendix with positive margins do not always indicate recurrence, making conservative management a reasonable option.^32^ However, some studies indicate that a positive surgical margin is a significant risk factor for recurrence.^29^

Most ANs are comprised of primary epithelial neoplasms and NETs, while secondary ANs is uncommon. A diagnosis of secondary ANs was established when the appendix was either directly infiltrated by a primary malignancy or affected through metastatic spread.^2,33^

In this study, out of all ANs, 52 (91.22%) cases were primary, while 3 (5.26%) cases were secondary. Two of the secondary cases were adenocarcinomas originating from cecal primaries, and one case was a mixed adeno-neuroendocrine carcinoma involving the terminal ileum and caecum, with secondary extension to the appendix. Two lymphoma cases with an indistinct site of origin and widespread involvement of adjacent structures were classified as unclassified appendiceal neoplasms.

This single-center retrospective study is limited by incomplete clinical staging data through imaging, poor documentation and lack of molecular profiling. Advanced treatments like CRS and HIPEC were unavailable, and the absence of long-term follow-up prevented assessment of recurrence and survival outcomes.

This study benefits from over a decade of data collected from a tertiary care center, ensuring a broad and diverse case pool. HPE reports were available for all cases, allowing accurate classification. The study also captures a wide spectrum of appendiceal neoplasms, including several rare variants, enhancing its clinical relevance.

## CONCLUSION

The incidence of appendiceal neoplasms has been increasing, particularly among older adults, with mucinous tumors being the most common, reflecting a shift toward epithelial origins. Common presentations included acute appendicitis, right iliac fossa mass, and chronic pain, while CT remains the preferred diagnostic modality. Appendectomy was sufficient in most cases, with ileocecal resection and hemicolectomy required selectively, and revision surgery was rarely needed. Perioperative outcomes were excellent, though surgical site infection continues to be the most frequent postoperative complication.

## Data Availability

The data are available from the corresponding author upon reasonable request.
